# Benign retropneumoperitoneum developed after endoscopic sphincterotomy and large balloon dilation of biliary sphincter for removal of large biliary stones: a case report

**DOI:** 10.1186/1757-1626-1-279

**Published:** 2008-10-28

**Authors:** Panagiotis Katsinelos, Grigoris Chatzimavroudis, Ioannis Pilpilidis, Christos Zavos, Georgia Lazaraki, Dimitris Tzilves, George Paroutoglou, Jannis Kountouras

**Affiliations:** 1 Department of Endoscopy and Motility Unit, Central Hospital, Thessaloniki, Greece; 2 Department of Gastroenterology, Second Department of Internal Medicine, Ippokration Hospital, Aristotle University of Thessaloniki, Thessaloniki, Greece

## Abstract

Biliary endoscopic sphincterotomy (ES) followed by biliary orifice dilation (BOD) with large-diameter balloons (> 12 mm) is a relative new technique for extraction of large biliary stones. However, the safety and the potential complications of this combined technique are not known yet. We present a patient who developed benign retroperitoneum after ES plus BOD with large-diameter balloon for removal of a large biliary stone, which was successfully treated conservatively. To the best of our knowledge this is the first report of such a complication after introduction of this method to clinical practice.

## Background

Biliary endoscopic sphincterotomy (ES) and stone extraction are considered as a standard therapy for treatment of biliary duct stones [1,2]. However, removal of large bile duct stones can be challenging in certain situations, such as after gastric bypass surgery or in the presence of periampullary diverticulum, stones located centrally to strictures and impacted stones [3,4]. The introduction by Ersoz et al [5] of ES followed by biliary orifice dilation (BOD) with large-diameter balloons (> 12 mm) for extraction of large biliary stones was rapidly adopted with enthusiasm by other biliary endoscopists [3,6,7]. However, the safety and the potential complications of this new combined technique are not known yet.

We present a patient who developed benign retroperitoneum after ES plus BOD with large-diameter balloon (12 mm in diameter) for removal of a large biliary stone. To the best of our knowledge this is the first report of such a complication after introduction of this method to clinical practice.

## Case presentation

**Figure 1:**
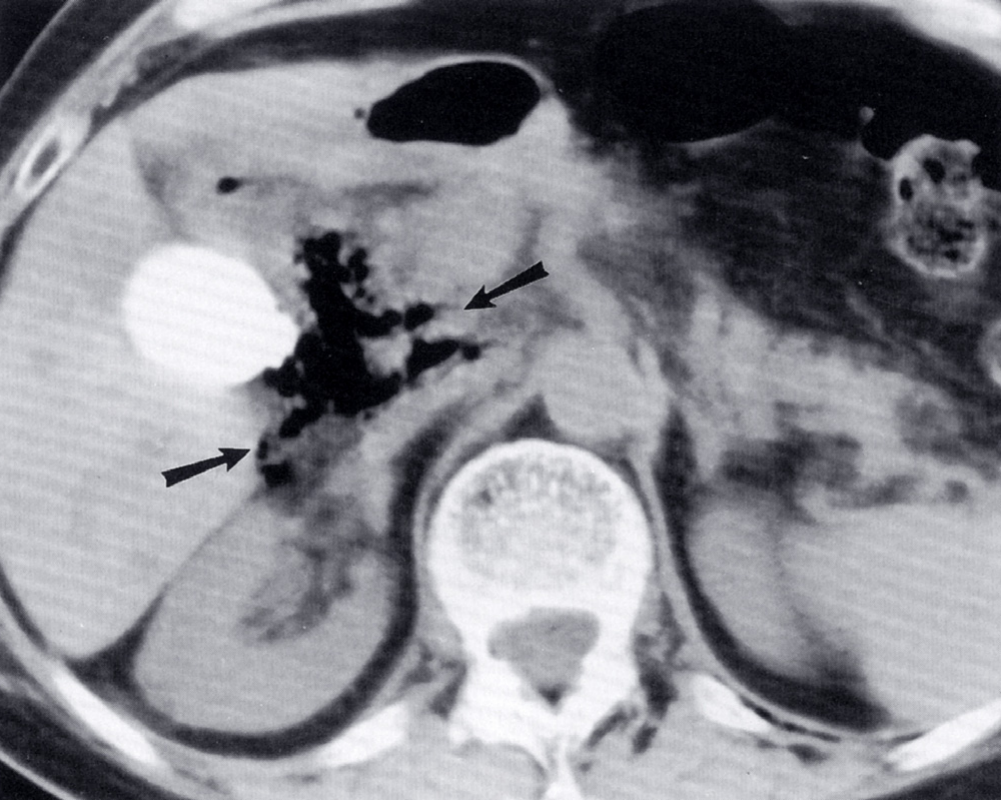
Radiograph showing air (arrow) in the retroperitoneal space.

A 78-year-old man with choledocholithiasis was referred to our department for ES and removal of biliary stones. On ERCP, the papilla was small without visible intramural course of common bile duct (CBD) in the duodenal wall. Cholangiography showed two large stones with diameters of 13 and 15 mm, respectively, in a dilated CBD (diameter of 16 mm). We performed the maximum possible, in length, ES, followed by mechanical lithotripsy and extraction of almost all the fragmented parts of stones. A relatively large piece of stone was impossible to be extracted by the balloon or captured by the lithotriptor. The biliary sphincter was dilated with a wire-guided balloon (CRE, Microvasive, USA) resulting in easy removal of the stone. On the afternoon the patient complained for a dull abdominal pain in the right upper quadrant but was afebrile without leucocytosis and there was no sign of peritoneal irrigation. An abdominal radiograph showed the presence of free air in the retroperitoneum (Fig. 1). An abdominal computed tomography (CT) revealed retropneumoperitoneum and aerobilia; however there was no evidence of contrast leakage from the bowel or biliary tract or retroperitoneal fluid collection. The patient was managed conservatively with intravenous fluid replacement, bowel rest and systemic antibiotics' administration. The abdominal pain was decreased gradually, while the patient continued to remain afebrile without leucocytosis. He was discharged 5 days after the procedure and a repeated abdominal CT, performed one month later, demonstrated complete absorption of retroperitoneal air.

## Discussion

ES with a large incision may be effective for extraction of large or difficult CBD stones; however, a large incision has a higher risk of perforation and probably a higher risk of bleeding [8,9]. Silent retroperitoneal air is not uncommon after ES and manipulation of papillary area [9-11]. Most of the symptomatic patients with retroperitoneal air are treated conservatively, but the risk of severe complications still exists [9-11]. Persistent abdominal pain and systemic toxic signs with leucocytosis should alert the management team, because continuous retroperitoneal leak of bile or duodenal juice leads to catastrophic results. Early surgical intervention, with extensive exploration, drainage and closure of the perforation, is generally indicated once severe retroperitoneal perforation is suspected [9-11].

To overcome the limitations of ES with large incision and endoscopic papillary balloon dilation (high rate of post-procedure pancreatitis and frequent use of mechanical lithotripsy), BOS after small or moderate ES has been advised as an effective method for retrieving large biliary stones without the use of mechanical lithotripsy [3,6,7].

Patients in whom bile duct stones cannot be removed because of a tapered distal bile duct and patients with large, square or barrel-shaped stones would benefit from this procedure.

The most common complications of this procedure are mild cholangitis, pancreatitis, bleeding and perforation. Complications occurred in 15.5% of patients in one study [6], with most of them (10.3%) being mild and self-limited. Moderately severe bleeding due to ES developed in three patients (5.2%), all of whom recovered without the need for surgery. Perforation did not occur in any patient who underwent dilation with a large diameter balloon. Mild pancreatitis developed in two patients (3.4%). Theoretically, the risk of pancreatitis by large balloon dilation after minimal or moderate sphincterotomy is less than balloon dilation alone. It is probable that after ES the force exerted by balloon dilation is directed mostly toward the common bile duct than the pancreatic orifice. Minimal or moderate ES before large balloon dilation might decrease the risk of pancreatitis as compared to dilation alone.

Overt perforations at the level of the major papilla after combined ES plus BOD have been rarely reported [3,6,7], suggesting that initial sphincterotomy incision followed by the stretching and tearing effect of the forcible dilation is a safe way to provide the maximum exit for stones even when balloons of up to 18 mm are used. Endoscopic papillary dilation should be performed slowly with a large balloon (maximum of 20 mm in diameter) to match the size of the bile duct. The use of balloons having diameter larger than common bile duct size can lead to increased incidence of perforation. Approximately 1 minute of balloon dilation is considered sufficient. We believe that patients with distal CBD stenosis, a narrow CBD or non-visible intramural course of CBD are at risk of perforation after balloon dilation. Therefore, it seems prudent to avoid excessive dilation in patients with these characteristics.

## Conclusion

The presence of air in the retroperitoneum is not always a detrimental complication of ES plus BOD and can be successfully treated conservatively in selected cases.

## Competing interests

The authors declare that they have no competing interests.

## Authors' contributions

PK performed the endoscopic sphincterotomy and was a major contributor in writing the manuscript. GC, IP and CZ analyzed and interpreted the patient data and were contributors in writing the manuscript. GL, DT and GP reviewed the relative literature. JK was major contributor in revising the manuscript critically for important intellectual content. All authors read and approved the final manuscript.

## Consent

Written informed consent was obtained from the patient for publication of this case report and accompanying images. A copy of the written consent is available for review by the Editor-in-Chief of this journal.
